# Comparison of non-intubated and intubated video-assisted thoracoscopic surgeries of major pulmonary resections for lung cancer—a meta-analysis

**DOI:** 10.1186/s12957-021-02181-x

**Published:** 2021-03-23

**Authors:** Wenfei Xue, Guochen Duan, Xiaopeng Zhang, Hua Zhang, Qingtao Zhao, Zhifei Xin, Jie He

**Affiliations:** Department of Thoracic Surgery, Hebei Province General Hospital, No 348, Heping Road West, Xinhua District, Shijiazhuang, 050000 China

**Keywords:** Thoracic surgery, Non-intubated anesthesia, Major lung resection, Lobectomy, Segmentectomy, Spontaneous breathing, Meta-analysis

## Abstract

**Objective:**

The aim of this study was to compare the safety feasibility and safety feasibility of non-intubated (NIVATS) and intubated video-assisted thoracoscopic surgeries (IVATS) during major pulmonary resections.

**Methods:**

A meta-analysis of eight studies was conducted to compare the real effects of two lobectomy or segmentectomy approaches during major pulmonary resections.

**Results:**

Results showed that the patients using NIVATS had a greatly shorter hospital stay and chest-tube placement time (weighted mean difference (WMD): − 1.04 days; 95% CI − 1.50 to − 0.58; *P* < 0.01) WMD − 0.71 days; 95% confidence interval (CI), − 1.08 to − 0.34; *P* < 0.01, respectively) while compared to those with IVATS. There were no significant differences in postoperative complication rate, surgical duration, and the number of dissected lymph nodes. However, through the analysis of highly selected patients with lung cancer in early stage, the rate of postoperative complication in the NIVATS group was lower than that in the IVATS group [odds ratio (OR) 0.44; 95% CI 0.21–0.92; *P* = 0.03, *I*^2^ = 0%].

**Conclusions:**

Although the comparable postoperative complication rate was observed for major thoracic surgery in two surgical procedures, the NIVATS method could significantly shorten the hospitalized stay and chest-tube placement time compared with IVATS. Therefore, for highly selected patients, NIVATS is regarded as a safe and technically feasible procedure for major thoracic surgery. The assessment of the safety and feasibility for patients undergoing NIVATS needs further multi-center prospective clinical trials.

## Introduction

Since video-assisted thoracoscopic surgery (VATS) with the double-lumen endotracheal tube and endobronchial blocker for one-lung ventilation was firstly used for the major pulmonary resections in 1992, it has been commonly adopted by thoracic surgeons due to its minimal invasive characteristic to patients [1]. Currently, this technique has been widely used for major pulmonary resections [2, 3] and intubated one-lung ventilation is a major milestone in thoracic surgical procedures [4]. However, the complications of general anesthesia with intubation cannot be neglected, such as intubation-related airway injury, ventilation-induced lung injury, residual neuromuscular blockade, and postoperative nausea and vomiting [5]. Tracheobronchial rupture may lead to a mortality rate as high as 22% [6]. Therefore, a variety of VATS were developed in the past decades to maintain spontaneous ventilation and reduce the adverse effects of general anesthesia [7, 8]. The utilization of VATS with spontaneous ventilation in mediastinal biopsies [9], metastatic tumors [10], bullectomy [11], empyema thoracic [12], pulmonary biopsies [13], pleural effusion [14], spontaneous pneumothorax [15], and non-anatomical resections has determined that this technique is a safe, efficient, and feasible technique for thoracic surgery [10].

Recently, non-intubated anesthesia has been gradually developed to minimize the damages of VATS. This makes the surgeons easier to use the non-intubated video-assisted thoracoscopic surgeries (NIVATS) in the anatomical lung resection [8]. Although there were many advantages for NIVATS with one-lung spontaneous ventilation than IVATS with mechanical ventilation, there are few papers to systematically compare the differences in NIVATS and IVATS in terms of safety and feasibility to patients during their major pulmonary resections.

## Material and methods

### Data collection

The keywords “non-intubated or non-tracheal intubation,” “awake or wake,” “video-assisted thoracoscopic surgery or VATS,” “regional anesthesia or local anesthesia” were combined with one another and entered into the Google Scholar, OVID, PubMed, Embase, and Cochrane library to identify relevant studies published before February 2020 for the meta-analysis. Studies had to meet the following criteria to be included in the analysis: (1) a randomized design was used; (2) observational studies comparing non-intubated VATS under local or regional anesthesia (experimental group) with radical intubated VATS under general anesthesia (control group) in patients for thoracic surgery; (3) the patients received the major surgical procedures including lobectomy and anatomical segmentectomy under VATS; (4) sufficient data could be obtained for the estimation of weighted mean differences (WMD) or odds ratios (OR); (5) replicated samples (or treatments) were considered. To avoid the specific selection of studies, these relatively accurate data without randomized organization should not be simply ignored and could also be included in the meta-analysis with an evaluation with the Newcastle-Ottawa Scale (NOS) [16]. To well illustrate the objective of this study, the following studies should not be considered as meta-data of this work: (1) without the comparison of non-intubated VATS with intubated VATS for thoracic surgery; (2) patients in both (control and experimental) groups received different surgical procedures; (3) minor pulmonary resections, such as wedge resection, metastasectomy, bullectomy, and non-anatomical resections; (4) letters to editors, case reports, meta-analysis, and reviews could not be considered.

A total of 8 published articles [17–24] were selected from 298 potential literature with the proposed paper selection criteria and they were listed in Tables 1 and 2. Specifically, there were 1 RCT study and 7 retrospective studies and a total of 970 patients were finally available for this study since they underwent the major pulmonary resections. The raw data consisted of surgical duration, hospitalized stays, lymph node numbers, chest-tube placement time, the volume of drainage, and rate of postoperative complications. There were nonfatal complications reported in these studies, including prolonged air leaks, atrial fibrillation, pneumonia, and atelectasis.

**Table 1:** Characteristics of the studies included in our meta-analysis

Author	Year	No. of case/control	Indications	Tumor size(cm)		Study design	Quality assessment
				Case	Control		
Jiang Bo et al.	2017	30/30	Lobectomy	2.08 ± 0.41	2.24 ± 0.42	Retrospective review	NOS:6
Zhihua Guo et al.	2016	48/92	Segmentectomy	NR	NR	Retrospective review	NOS:7
Jin-shing Chen et al.	2011	30/30	Lobectomy	2.1 ± 1.2	1.9 ± 0.7	Retrospective review	NOS:6
Jun Liu et al.	2016	20/20	Segmentectomy	1.0 ± 0.4	1.6 ± 1.1	Retrospective review	NOS:7
Jun Liu et al.	2016	116/116	Lobectomy	2.4 ± 1.3	2.5 ± 1.2	Retrospective review	NOS:7
Jun Liu et al.	2014	26/30	Lobectomy	NR	NR	RCT	Jadad score:2
Zeead M.AlGhamdi et al.	2018	30/30	Lobectomy	NR	NR	Retrospective review	NOS:7
Chun-Yu Wu et al.	2013	36/48	Lobectomy	2.9 ± 1.6	3.0 ± 1.8	Retrospective review	NOS:7
Lan Lan et al.	2018	119/119	Lobectomy	NR	NR	Retrospective review	NOS:7

*RCT* randomized controlled trial, *NOS* Newcastle-Ottawa scale, *NR* not report

**Table 2:** Main data extracted from the studies

Author(year)	Global in operating room time (min)^a^		Hospital stays (days)^a^		Postoperative complications^b^		Surgical duration (min)^a^		Lymph node dissection number^a^		Total fluid administration (ml)^a^		Postoperative chest drainage (days)^a^	
	Case	Control	Case	Control	Case	Control	Case	Control	Case	Control	Case	Control	Case	Control
Jiang Bo 2017 [21]	NR	NR	6.67 ± 1.42	7.53 ± 1.61	1/30	2/30	74.83 ± 48.38	77.17 ± 23.26	8.67 ± 2.34	8.43 ± 2.33	297.3 ± 249.4	318 ± 190.7	2.17 ± 1.09	3.06 ± 1.19
Zhihua Guo 2016 [18]	NR	NR	6.04 ± 3.60	7.83 ± 5.89	4/48	14/92	168.6 ± 57.6	148.2 ± 52.2	8.06 ± 6.22	8.02 ± 4.31	383.46 ± 47.54	626.98 ± 117.18	2.25 ± 1.36	3.16 ± 3.93
Jin-shing Chen 2011 [24]	229.3 ± 43.7	223.2 ± 46.6	5.9 ± 2.2	7.1 ± 3.2	3/30	10/30	161.9 ± 37.4	161.3 ± 41.4	13.8 ± 6.0	14.0 ± 6.0	NR	NR	3.6 ± 1.7	5.0 ± 4.0
Jun Liu 2016 [20] segment	NR	NR	6.0 ± 1.2	8.3 ± 4.3	3/20	3/20	152.5 ± 34.8	158.3 ± 48.8	7.8 ± 5.4	6.4 ± 5.3	354.5 ± 244.8	723.0 ± 717.4	2.6 ± 1.2	4.3 ± 7.2
Jun Liu 2016 [20] lobectomy	NR	NR	7.4 ± 2.0	8.6 ± 4.1	10/116	12/116	177.8 ± 43.0	182.0 ± 55.5	17.2 ± 9.1	15.7 ± 9.5	607.4 ± 378.8	766.7 ± 638.2	3.2 ± 2.6	3.5 ± 2.4
Jun Liu 2014 lobectomy [17]	NR	NR	NR	NR	NR	NR	NR	NR	NR	NR	NR	NR	NR	NR
Zeead M. AlGhamdi 2018 [22]	NR	NR	6.9 ± 3.8	7.6 ± 5.3	6/30	6/30	130.9 ± 30.1	146.0 ± 47.4	12.6 ± 6.0	18.0 ± 7.4	NR	NR	5.6 ± 7.0	5.4 ± 5.4
Chun-Yu Wu 2013 [23]	**247.9 38.5**	**276.6** ± **76.1**	**6.7** ± **3.3**	**7.2** ± **3.5**	9/36	17/48	**184.6** ± **32.3**	**212.6** ± **77.3**	**13.1** ± **7.7**	**15.5** ± **8.1**	**1326.7** ± **507.0**	**1750.0** ± **465.3**	NR	NR
Lan Lan 2018 [19]	NR	NR	NR	NR	41/119	240119	175.63 ± 55.67	217.64 ± 59.71	NR	NR	2105.04 ± 520.24	1822.29 ± 536.64	NR	NR

*NR* not reported

^a^Expressed as mean ± standard deviation

^b^Expressed as number of patients with complications/number of all patients without complications

### Data screening

**Fig. 1:**
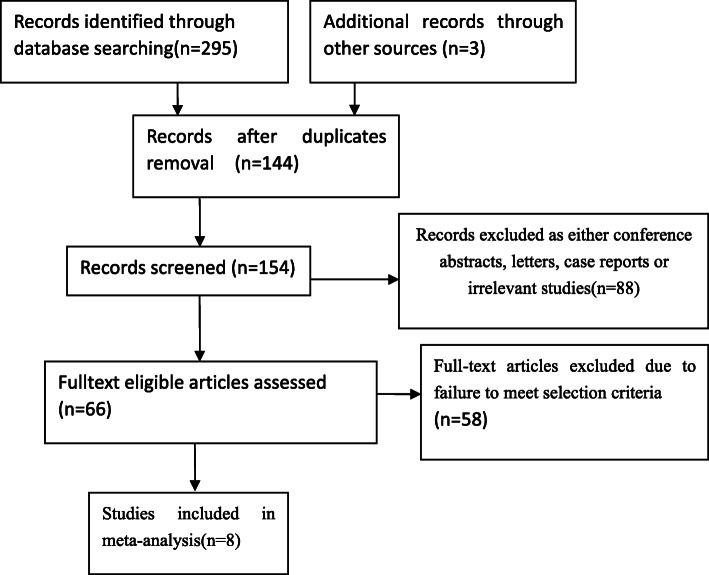
Search strategy

**Fig. 2:**
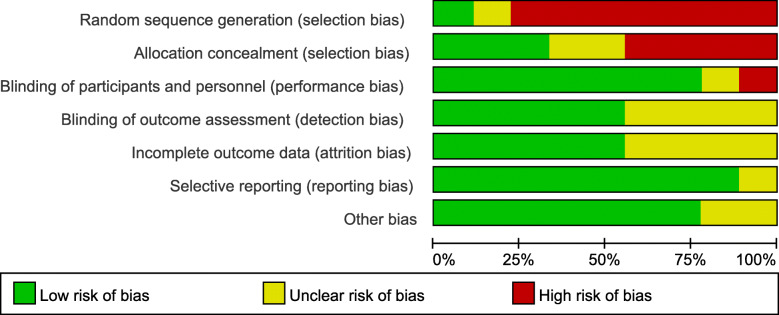
Risk of bias graph: a review of authors’ judgements about each risk of bias item presented as percentages across all included studies

**Fig. 3:**
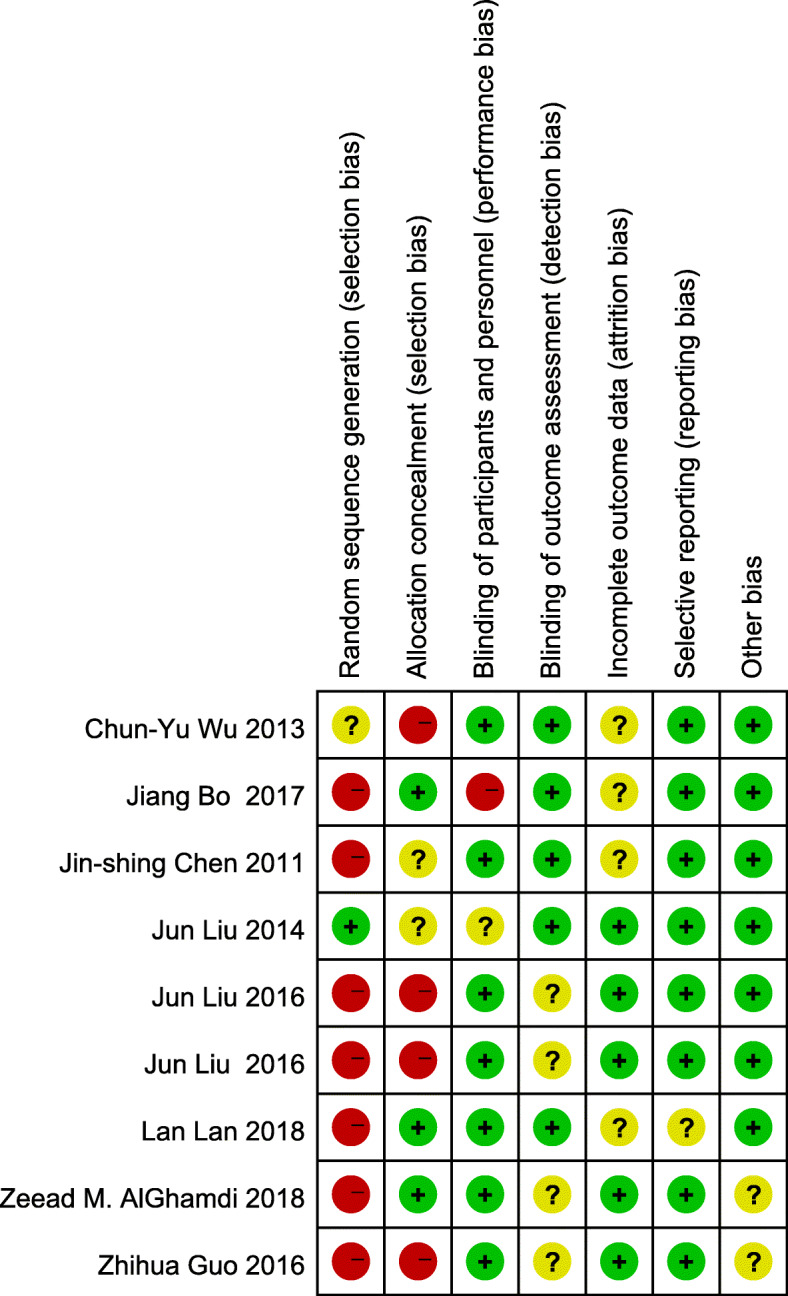
Risk of bias summary: a review of authors’judgements about each risk of bias item for each included study. The symbols “+”,“−”and“?”represent low risk of bias, high risk of bias and uncertain of bias, respectively

The data screening was conducted independently by two authors to extract the eligible mate-data for this research. When discrepancies appeared during the data selection process, the corresponding author would make the final adjudication to make sure that the extracted data were carefully retrieved from these studies (Fig. 1). According to the Cochrane Collaboration’s standard, the quality of each selected study was assessed to avoid the risk of bias [25] and this evaluation was made with the Jadad scale, which refers to randomization (0–2 points), blinding of the studies (0–2 points), and withdrawals (0–1 point). The studies are regarded as high quality while the score points are not lower than 3. Another method of Newcastle-Ottawa Scale (NOS) was also used to evaluate the meta-data quality with non-randomized studies [16]. Three important factors were considered in this evaluation, including patient selections, comparability of the study groups, and exposure. Assigning each study with a score of 0–9 (allocated as stars), the high-quality study was defined as a study with a quality score star not lower than 6. These studies were generally of high quality according to the Jadad scales and NOS. The bias risk summary was shown in Figs. 2 and 3. There was no significant difference in publication bias based on the Begg’s and Egger’s tests and the selected studies were of low risk.

### Statistical analysis

**Fig. 4:**
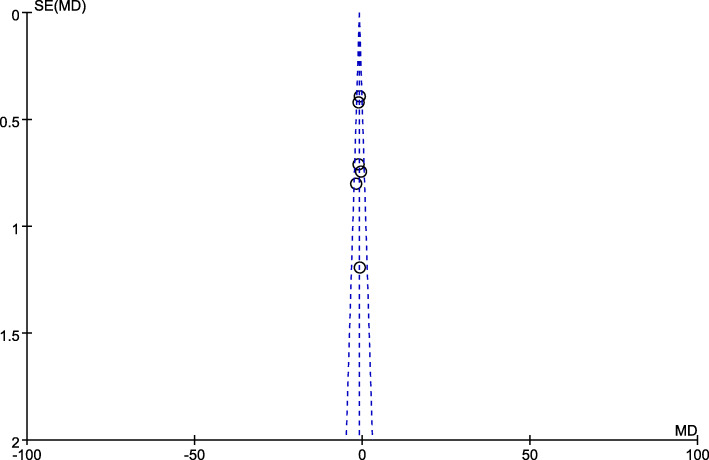
Funnel plot of the included studies for overall analysis of hospital stays

The meta-analysis was conducted using Review Manager 5 software (RevMan-5, Cochrane Community, London, UK). Statistical heterogeneity was estimated by Higgins *I*^2^, which represents the total variation percentage among the studies. A fixed-effect model (Mantel–Haenszel method) was used to pool homogeneous studies while the I^2^ was less than 50%. Otherwise, the random-effect model (DerSimonian-Laird was used. Estimation of potential publication bias was conducted by the funnel plot and the asymmetry was assessed by Begg’s test and Egger’s test [26] (Fig. 4). The statistical significance was appointed once the *P* value was lower than 0.05.

## Results

**Fig. 5:**
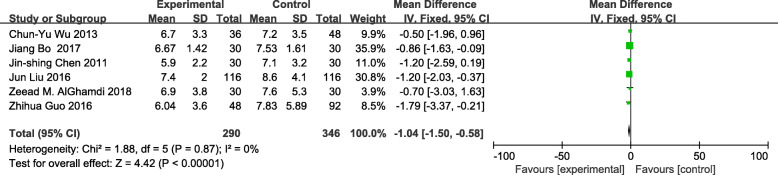
Forest plot of hospital stay for the non-intubated group vs. the intubated group. CI: confidence interval; IV: inverse variance; SD: standard deviation

**Fig. 6:**
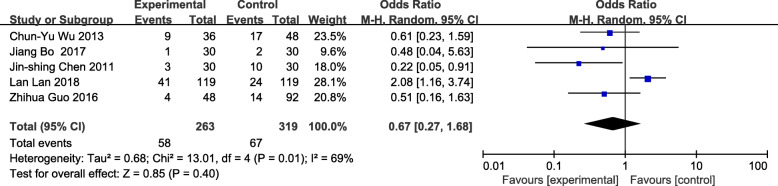
Forest plot of postoperative complication rate for the non-intubated group vs. the intubated group

**Fig. 7:**
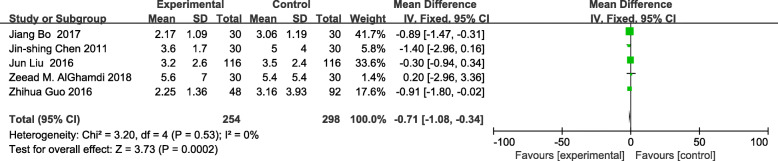
Forest plot of duration of chest-tube placement for the non-intubated group vs. the intubated group

**Fig. 8:**
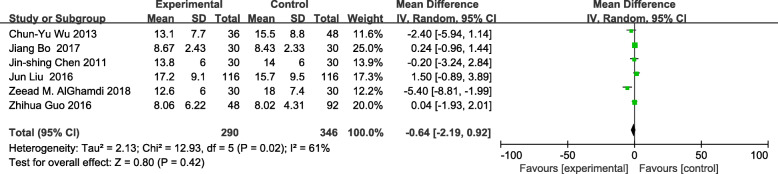
Forest plot of lymph node numbers for the non-intubated group vs. the intubated group

**Fig. 9:**
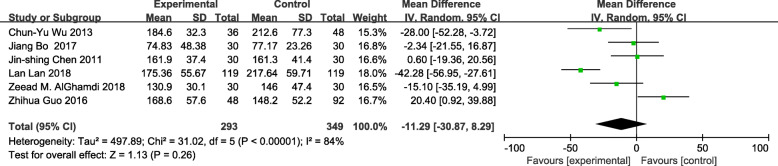
Forest plot of surgical duration for the non-intubated group vs. the intubated group

**Fig. 10:**
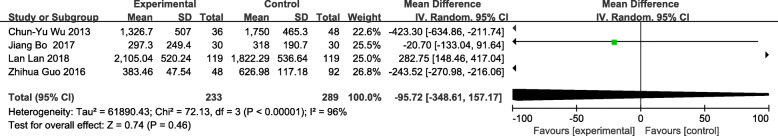
Forest plot of volume of drainage for the non-intubated group vs. the intubated group

**Fig. 11:**
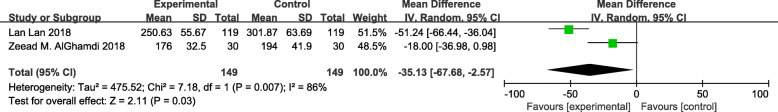
Forest plot of global in-operating room time for the non-intubated group vs. the intubated group

**Fig. 12:**
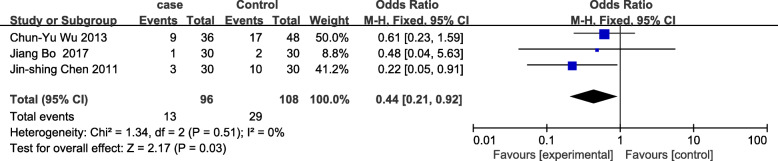
Forest plot of postoperative complication rate for highly selected patients for the non-intubated group vs. the intubated group

The meta-analysis of eligible studies was conducted to compare the feasibility and safety of NIVATS to IVATS under loco-regional anesthesia for major thoracic surgery. In this study, only eight studies were thoroughly concluded due to the duplicated data. Results showed that NIVATS significantly shortened the hospitalized stay compared to VATS (WMD − 1.04 days; 95% CI − 1.50 to − 0.58; *P* < 0.01) (Fig. 5). The rate of postoperative complication was analyzed based on five studies and no significant differences were observed [OR 0.67; 95% CI 0.27–1.68; *P* = 0.40] (Fig. 6). But the duration of chest-tube placement was greatly shortened with NIVATS than those with IVATS (WMD − 0.71 days; 95% CI − 1.08 to − 0.34; *P* < 0.01) (Fig. 7). There were no significant differences in the number of dissected lymph nodes (WMD − 0.64; 95% CI − 2.19 to 0.92; *P* = 0.42) (Fig. 8), surgical duration (WMD − 11.29 min; 95% CI − 30.87 to 8.29; *P* = 0.26) (Fig. 9), and volume of drainage (WMD − 95.72; 95% CI − 348.61 to 157.17; *P* = 0.46) (Fig. 10) between NIVATS and IVATS. Only two studies reported global in-operating room time, and it was concluded that the global in-operating room time was much shorter for patients with INVATS under loco-regional anesthesia than those with IVATS under general anesthesia [random effects WMD − 35.13; 95% CI − 67.68 to − 2.57; *P* < 0.05; *I*^2^ = 86%] (Fig. 11). Through the highly selected patients with lung cancer in the early stage, we found that the rate of postoperative complications was lower for the patients in the NIVATS group than those in IVATS group [OR 0.44; 95% CI 0.21–0.92; *P* = 0.03; *I*^2^ = 0%] (Fig. 12).

**Fig. 13:**
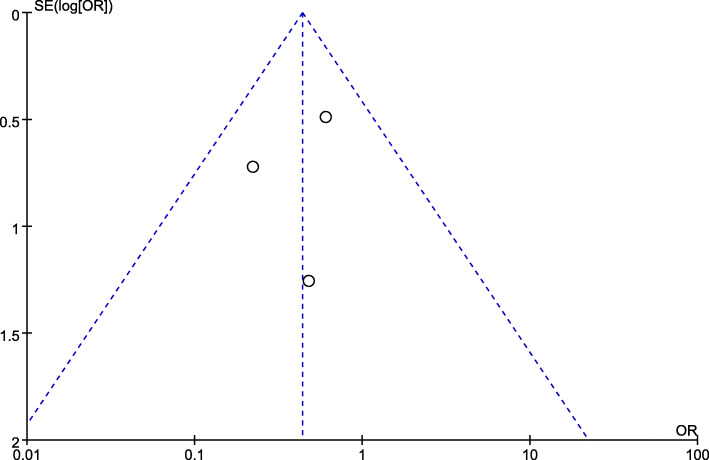
Funnel plot illustrates the meta-analysis of postoperative complication rate. SE, standard error

A funnel plot estimating the precision of the trials (plots of the logarithm of the OR for efficacy against sample size) was examined for asymmetry to determine publication bias (Fig. 13). It showed that the outcomes were similar regardless of whether fixed-effects models or random-effects models utilization.

## Discussion

With the development of lung separation technology and the application of double-lumen endotracheal, which can provide excellent exposure and a quiet surgical environment for thoracic surgeons, the intubated VATS with general anesthesia has been proposed to be a mandatory surgical procedure in recent years, whereas the complications associated with mechanical ventilation or intubation-related cannot be effectively avoided [27, 28]. Due to the pursuit of minimally invasive surgical strategies in thoracic surgeons, thoracoscopic surgery without tracheal intubation has been applied to patients with pleural or peripheral lung diseases [29]. However, it is still unclear that the NIVATS is adopted or not to treat the patients with lobectomy and segmentectomy. In general, major pulmonary resections to non-intubated patients are significantly different from the performance of minor procedures. The potential risk of major bleeding in the pulmonary hilum during a lobectomy to a patient with spontaneous ventilation is higher than the risk of a surgical complication during a wedge or lung biopsy. The performance of a lobectomy with mediastinal lymph node dissection by VATS or the intense pulmonary manipulation during segmentectomy might trigger coughing in spontaneously breathing patients. Previous studies demonstrated that intrathoracic vagal blockade to abolish the cough reflex was effective during non-intubated lobectomy and segmentectomy [24, 30]. The combination with epidural anesthesia and the phrenic and vagus nerves blockade provided a stress-free surgery [31]. In order to ensure patient safety, it is inevitable that spontaneously breathing converts to general anesthesia with tracheal intubation [32]. Chen et al. [24] reported that the rate of conversion to intubated-single lung ventilation was 10%, because of persistent hypoxemia, poor epidural anesthesia, and bleeding from dividing pleural adhesions and incomplete fissure. Guo Z et al. [18] observed that patients (2.1%) required conversion to intubated single-lung ventilation because of vigorous mediastinal movement.

The safety and feasibility of NIVATS were investigated for the major pulmonary resections. Results showed that there were no statistically significant differences in postoperative complication rate. So far, many studies had reported that the NIVATS procedure is a safe, effective, and feasible technique for the minor pulmonary resection to minimize the trauma, quick recovery, and low rate of postoperative complication. The discrepancy on the major surgical procedure with NIVATS still existed. AlGhamdi et al. [22] and Wu et al. [23] reported that no significant differences were found in complication rate between NIVATS and IVATS methods. However, Chen et al. [24] reported that non-intubated patients had a lower non-complication rate, which suggested that non-intubated thoracoscopic lobectomy was feasible and safe. Therefore, this meta-analysis provided more evidences to establish the short-term feasibility and safety profile of non-intubated VATS under loco-regional anesthesia for major thoracic surgery. Through this meta-analysis, it was found that the most important factors were the surgeon anesthetist and their different skill levels, which could significantly determine the duration of the operation and postoperative recovery time. Another factor was the patient selection. The results indicated that the more highly selected, the more superiority might be verified. Therefore, in order to decrease the risk of emergency intubation and complications, the proper patient should be selected to use INVATS, especially at the beginning of the learning level.

There are still some limitations in this meta-analysis. Firstly, more publications should be considered in future meta-analysis studies to make the results more convincing. Secondly, most of these studies were derived from medical centers located in south China, which may not represent the general situations. Thirdly, the analysis was conducted by the random-effects model, which could weaken our analytical power due to the significant heterogeneity. Therefore, further studies are required to evaluate the safety and feasibility of NIVATS in major pulmonary resections.

## Conclusions

Based on the results obtained in this meta-analysis, there were no significant differences between NIVATS and IVATS in postoperative complication rate of major thoracic surgery. However, it was obvious that the NIVATS utilization could significantly shorten the chest-tube placement duration and patients’ hospitalized staying period compared to IVATS. The main reason may be due to the avoidance of intubation, mechanical ventilation, muscle relaxants, and routine use of perioperative epidural anesthesia in these patients with NIVATS. Overall, NIVATS in major thoracic surgery is a safe and technically feasible procedure and it can be used to replace the IVATS to some extent. Further studies are required to be conducted to compare these two methods in long-term clinical experiments.

## Data Availability

All the data used in this work are available from the corresponding author upon reasonable request.
